# Emergency department staff compassion is associated with lower fear of enacted stigma among patients with opioid use disorder

**DOI:** 10.1111/acem.14970

**Published:** 2024-06-17

**Authors:** Savannah Steinhauser, Rachel Haroz, Iris Jones, William Skelton, Brian M. Fuller, Michael B. Roberts, Christopher W. Jones, Stephen Trzeciak, Brian W. Roberts

**Affiliations:** ^1^ The Department of Emergency Medicine, Cooper University Health Care (CUHC) Cooper Medical School of Rowan University (CMSRU) Camden New Jersey USA; ^2^ The Department of Emergency Medicine Division of Toxicology and Addiction Medicine, CUHC/CMSRU Camden New Jersey USA; ^3^ Cooper Center for Healing, CUHC/CMSRU Camden New Jersey USA; ^4^ Department of Behavioral Medicine CUHC/CMSRU Camden New Jersey USA; ^5^ Division of Critical Care Medicine, Departments of Emergency Medicine and Anesthesia Washington University School of Medicine St. Louis Missouri USA; ^6^ Institutional Research and Outcomes Assessment Philadelphia College of Osteopathic Medicine Philadelphia Pennsylvania USA; ^7^ The Department of Medicine CUHC/CMSRU Camden New Jersey USA

**Keywords:** compassion, emergency department, empathy, opioid use disorder, stigma, substance use disorder

## Abstract

**Objectives:**

Fear of enacted stigma (fear of discrimination or being treated unfairly) is associated with decreased health care–seeking behaviors among patients with opioid use disorder (OUD). We sought to describe the prevalence of fear of enacted stigma among patients presenting to the emergency department (ED) with OUD and to test whether experiencing greater compassion from ED staff is associated with lower fear of enacted stigma.

**Methods:**

We conducted a cross‐sectional study in the ED of an academic medical center between February and August 2023. We included adult patients with OUD presenting to the ED and assessed patient experience of compassion from ED staff using a previously validated 5‐item compassion measure (score range 5–20). The primary outcome measure was fear of enacted stigma in the ED, measured using the validated 9‐item subscale of the Substance Abuse Self‐Stigma Scale (score range 9–45).

**Results:**

Of the 116 subjects enrolled, 97% (95% confidence interval [CI] 91%–99%) reported some degree of stigma, with a median (interquartile range) score of 23 (16–31). In a multivariable model adjusting for potential confounders, patient experience of greater ED compassion was independently associated with lower fear of enacted stigma, β = −0.66 (95% CI −1.03 to −0.29), suggesting that every 1‐point increase in the 5‐item compassion measure score is associated with a 0.66‐point decrease in the fear of enacted stigma score.

**Conclusions:**

Among ED patients with OUD, fear of enacted stigma is common. Patient experience of compassion from ED staff is associated with lower fear of enacted stigma. Future research is warranted to test if interventions aimed at increasing compassion from ED staff reduce patient fear of enacted stigma among patients with OUD.

## INTRODUCTION

Opioid use disorder (OUD) is a major public health crisis that is expanding in the United States. The nationwide OUD epidemic is affecting individuals of every gender, race, and socioeconomic status.^1^ In 2022, over 8.9 million people age 12 and older in the United States had misused opioids in the past year and over 6.1 million people had an OUD.^2^ In recent years, rates of opioid overdose have accelerated, with over 80,000 overdose deaths in 2021.^3^, ^4^ Individuals with OUD are at high risk for experiencing acute medical emergencies such as overdose or infectious complications related to intravenous (IV) drug use, and many patients with OUD do not have preexisting established relationships with health care providers.^5^ As a result, chronic and subacute issues often go unattended, leading to individuals with OUD to use the emergency department (ED) for medical care. ED visits for opioid‐related complications have continued to increase over recent years, making the ED a vital setting for engaging patients and initiating long‐term treatment.^6^, ^7^, ^8^

Enacted stigma is the direct experience of social discrimination and being treated unfairly by others due to identification with a stigmatized group, which results in self‐stigma and feelings of shame.^9^, ^10^ Among patients with OUD, fear of enacted stigma is associated with decreased health care–seeking behaviors, putting patients at risk for adverse events.^11^, ^12^ Experiencing stigma during clinical encounters may in part contribute to the fact that only 11% of people with OUD are currently receiving medication for opioid use disorder (MOUD).^13^ Consequently, the National Institute of Drug Abuse (NIDA) strategic plan calls for the development of interventions to reduce the stigma that impedes access to health care for people with OUD.^13^ Understanding the factors associated with stigma is a critical component of addressing this significant public health crisis.

Health care provider compassion is a fundamental element of the therapeutic relationship; both patients and physicians consider compassion to be a vital part of high‐quality care.^14^, ^15^ Greater patient experience of clinician compassion lowers patients’ anxiety and distress,^16^ and among patients presenting to the ED for a medical emergency, greater ED compassion is associated with lower development of PTSD symptoms^17^ and better patient‐reported outcomes among trauma patients.^18^ Thus, compassion experienced by patients in the ED may be associated with less fear of enacted stigma.

Our study objectives were to (1) determine the degree of fear of enacted stigma experienced by patients presenting to the ED with OUD and (2) test the association between patient experience of compassion in the ED and fear of enacted stigma. We hypothesized that greater patient experience of compassion in the ED is associated with a decrease in fear of enacted stigma.

## METHODS

### Setting

We performed a cross‐sectional study at a single academic ED in the United States. All ED physicians are trained in initiation of MOUD in the ED, and patients are referred to a local addiction medicine clinic that accepts same‐day walk‐in visits or an opioid treatment program for patients initiated on methadone. The institutional review board at our institution approved this study, and all subjects provided written informed consent prior to participation. This study is reported in accordance with the Strengthening the Reporting of Observational Studies in Epidemiology Statement (Table S1)^19^ and the Checklist for Reporting Of Survey Studies (Table S2).^20^

### Participants

We included adult patients presenting to the ED with a history of OUD between February 2023 and August 2023. Inclusion criteria were: (1) age 18 years or older; (2) presenting as a patient to the ED; (3) history of OUD documented in the electronic medical record and/or presented to the ED for an opioid‐related complication (opioid overdose, opioid withdrawal, requesting MOUD administration or addiction rehabilitation placement); (4) patient‐reported active illicit opioid use and/or current participation in a MOUD program (methadone or buprenorphine); and (5) determined to be clinically sober at the time of enrollment by the treating ED physician. Exclusion criteria included: (1) found to have an acute psychiatric emergency (e.g., active suicidal ideation or psychosis); (2) unable to participate in the research questionnaires (i.e. history of dementia, critically ill); (3) previously participated in the study; (4) prisoner; and (5) non–English‐speaking. A convenience sample was obtained between the hours of 2 and 9 p.m., 7 days per week. During this time research academic associates (RAA) staff the ED. RAAs are volunteer premedical and postbaccalaureate students who received human‐subject protection training as well as training in principles of clinical research. Specific to this study, RAAs received didactic lectures and one‐on‐one training on screening, enrollment, and survey administration from the study investigators (CWJ and BWR). While in the ED, RAAs screened the electronic medical record, EPIC, on patient arrival to the ED for documented history of OUD as well as screened the ED track board for OUD‐related reason for ED visit.

### Data collection

At the completion of care by the ED clinician (i.e., either time of hospital admission or discharge home from the ED for patients not admitted to the hospital) RAAs approached potential subjects for enrollment. After written informed consent was obtained, subjects were given the research questionnaire in paper form. RAAs were instructed to inform the subjects that only the research team will have access to questionnaire answers; specifically, answers will not be shared with the hospital staff involved in their clinical care. After handing the subject the questionnaire, RAAs were instructed to leave the patients’ bedside to allow for privacy while filling out the questionnaire and to return in approximately 10 min to collect the form.

The research questionnaire assessed patient experience of ED staff compassion using the 5‐item compassion measure, a patient‐assessed measure of health care compassion (Figure S1). The 5‐item compassion measure has previously been psychometrically validated for use in the ED, outpatient, and inpatient settings and consists of 5 items measured on a 4‐point Likert scale (Figure S1).^21^, ^22^, ^23^ The 5‐item compassion measure was asked in regard to all ED staff, to assess the overall experience of compassion while in the ED. Potential scores range from 5 to 20 with higher scores indicating greater compassion. We also assessed for self‐reported access to shelter (i.e., In the last month, I most often slept … in a shelter or on the street; at a friend/family member's house; in an apartment/condo/home I rented; in an apartment/condo/home I own); number of ED visits in the past 30 days, prior or current use of methadone or buprenorphine, and number of years of opioid use. We collected data on ED wait time (time from ED arrival to time seen by a physician), length of ED stay (time from ED arrival to time leaving the ED), whether the patient was evaluated in a hallway bed versus a private room in the ED,^24^ and reason for ED visit. Reason for ED visit was further categorized as an opioid‐specific problem (i.e., opioid overdose, opioid withdrawal, requesting MOUD administration or addiction rehabilitation placement, or possible IV drug use–related infection [diagnosed with an infection during the ED visit and history of IV opioid use]) versus non–opioid‐related problem. Two independent reviewers (SS and BWR) reviewed the electronic medical records and questionnaire responses. The two reviewers adjudicated the reason for ED visit. In cases of disagreement, a plan for a third reviewer was in place, but was not needed. We abstracted demographics, comorbid conditions (i.e., Charlson Comorbidity Index, higher score predictive of greater 1‐year mortality [0 = none, 1–2 = low, 3–4 = moderate, ≥5 = high]),^25^ and ED crowding at the time of patient arrival using the National Emergency Department Over Crowding Study (NEDOCS) tool, from the medical record (NEDOCS >100 = overcrowded, >160 = severely overcrowded, and >200 = dangerously overcrowded).^26^

### Primary outcome measure

The primary outcome measure was fear of enacted stigma, measured at the completion of care by the ED clinician using the previously validated, 9‐item subscale of the Substance Abuse Self‐Stigma Scale.^9^ The fear of enacted stigma subscale has previously demonstrated good internal consistency, a reliable factor structure, and predicted correlations with established measures.^9^ The 9 items were measured on a 5‐point Likert scale referencing “how many people working in the emergency department do you think would react to you as described” (1, few people [0%–20%]; 2, some people [20%–40%]; 3, many people [40%–60%]; 4, most people [60%–80%]; 5, almost everyone [80%–100%]; Figure S2). Potential scores range from 9 to 45 with higher scores indicating greater fear of enacted stigma. The initial validation study found a mean (±SD) fear of enacted stigma score of 23.7 (±8.4) among patients with OUD.^27^ We entered all data into Research Electronic Data Capture (REDCap), a secure, web‐based application designed to support data capture for research studies,^28^, ^29^ and exported into Stata/SE 16.1 for Mac for analysis.

### Data analysis

#### Descriptive analyses

We report continuous variables as median and interquartile range (IQR) and categorical variables as frequencies and percentages. We tested the internal reliability of the 5‐item compassion measure and the fear of enacted stigma subscale using Cronbach's alpha. We summed the scores for each individual item to obtain a composite score for the 5‐item compassion measure (higher scores indicating greater compassion [score range 5–20]) as well as for the fear of enacted stigma subscale (higher scores indicating greater fear of enacted stigma [score range 9–45]). We display the full distribution of the composite 5‐item compassion measure and the fear of enacted stigma subscale in histogram form.

To assess whether patient or ED characteristics external to the patient–staff interaction were associated with patient experience of compassion, we used multivariable linear regression with the 5‐item compassion measure as the dependent variable and entered the following independent variables into the model: (1) female sex, (2) race—White versus non‐White, (3) ED wait time (min), and (4) ED clinician evaluation in a hallway bed versus a private room.

#### Primary analysis

For the primary analysis we used linear regression to test whether patient experience of compassion (i.e., 5‐item compassion measure composite score) is associated with patient fear of enacted stigma while in the ED. We then adjusted the model for potential confounders selected a priori:^30^ (1) age in years; (2) history of polysubstance use (history of alcohol, benzodiazepine, or other illicit drug(s) use in addition to opioids); (3) history of IV opioid use versus non‐IV opioid (e.g. inhalation) use;^11^ and (4) reported living in a shelter/on the street.

#### Sensitivity analyses

We performed a sensitivity analysis using structural equation modeling to perform a multivariable linear regression model using full information maximum likelihood estimation to allow patients with missing questionnaire data to be included.^31^

We performed a second sensitivity analysis entering an interaction term between patient experience of compassion and reason for ED visit for an opioid specific problem into the model. The objective of this sensitivity analysis was to test if the association between experience of compassion and fear of enacted stigma differed among patients presenting to the ED primarily for an opioid‐specific problem compared to patients with OUD presenting primarily for a non–opioid‐related problem.

For all models, we treated the ordinal variables of interest (i.e., patient experience of compassion and fear of enacted stigma) as continuous variables given wide range of possible scores and used conservative robust standard errors to estimate the 95% confidence intervals (CIs) to reduce the risk of Type I error.

#### Sample size calculation

Based on prior studies we assumed a fear of enacted stigma subscale SD of 7,^9^ a 5‐item compassion scale SD of 4,^21^, ^22^, ^23^ and an α = 0.05, we would require a minimum of 91 subjects to have an 80% power to detect a β ≤ −0.5 (every one‐point increase in the 5‐item compassion measure is associated with a 0.5 decrease in the fear of enacted stigma subscale). We chose a conservative effect size given no prior literature defining a clinically meaningful fear of enacted stigma score and the exploratory nature of this study.

## RESULTS

**FIGURE 1:**
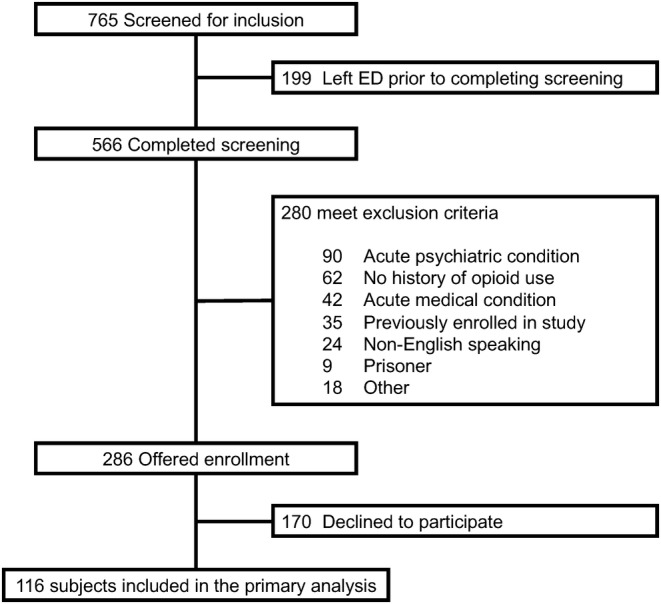
Study flow diagram.

A total of 116 subjects were enrolled and included in the study (Figure 1). Baseline data at the time of presentation to the ED for all subjects in the cohort is displayed in Table 1. The majority of patients used IV opioids (58.6%, 68/116) and most had a documented history of polysubstance use (85.3%, 99/116) (Table 1). Twenty (17.2%) patients presented for infectious symptoms, of which 65.0%, (13/20) used IV opioids, and overall, 50.0% (58/116) of patients presented to the ED for an opioid specific problem. Seventy eight per cent reported current or prior MOUD treatment (i.e. methadone or buprenorphine) (Table 2). The median [IQR] wait time was 56 (26–94) minutes and the majority of patients were evaluated in a hallway bed (78.6%, 91/116). The median [IQR] NEDOCS score was 175 [148–202], meaning the majority of patients presented when the ED was considered severely overcrowded or dangerously overcrowded. The median [IQR] ED length of stay was 6.6 (3.5–12.2) hours and 31.0% (36/116) of patients were admitted to the hospital from the ED.

**TABLE 1:** Baseline patient characteristics.

Variable	All Subjects *n* = 116
Age (years)	42 (35–54)
Female [*n* (%)]	42 (36.2)
Race [*n* (%)]	
White/Caucasian	70 (60.3)
Black/African American	39 (33.6)
Other	7 (6.0)
Hispanic ethnicity [*n* (%)]	13 (11.2)
Pre‐existing comorbidities [*n* (%)]	
Diabetes	20 (17.2)
Known coronary artery disease	5 (4.3)
Hypertension	45 (38.8)
Malignancy	6 (5.2)
Renal insufficiency	6 (5.2)
Pulmonary disease	27 (23.3)
Cerebral vascular disease	6 (5.2)
Congestive heart failure	8 (6.9)
Human immunodeficiency virus	4 (3.5)
Charlson comorbidity score [median (IQR)]	1 (0–2)
Substance use history [*n* (%)]	
IV opioids	68 (58.6)
Marijuana	65 (56.0)
Cocaine	75 (64.7)
Phencyclidine	14 (12.1)
Methamphetamine	13 (11.2)
Benzodiazepine	9 (7.8)
Alcohol	49 (41.4)
Reason for ED visit	
Opioid overdose	8 (6.9)
Opioid withdrawal	10 (8.6)
Requesting opioid rehabilitation placement	11 (9.5)
Requesting MOUD dose	17 (14.7)
Musculoskeletal	19 (16.4)
Infection	20 (17.2)
Cardiopulmonary	20 (17.2)
Gastrointestinal	19 (8.6)
Other	9 (7.8)

*Note*: Data are reported as median (IQR) or *n* (%).

Abbreviations: IQR, interquartile range; MOUD, medication for opioid use disorder.

**TABLE 2:** Self‐reported demographics.

Variable	All subjects *n* = 116
In the last month, I most often slept…	
In a shelter or on the street	31 (26.7)
At a friend/family members house	40 (34.5)
In an apartment/condo/home I rented	20 (17.2)
In an apartment/condo/home I own	13 (11.2)
Unknown	12 (10.3)
Prior to today, how many times in the last 30 days have you visited an emergency department? [median (range)]	1 (0 to 20)
	Missing data (*n* = 13)
Current or prior methadone use	63 (57.3)
	Missing data (*n* = 6)
Current or prior buprenorphine use	74 (67.3)
	Missing data (*n* = 6)
Approximately how many years have you been using opioids? [median (range)]	13 (<1 to 54)
	Missing data (19)

*Note*: Frequency (percent) reported unless otherwise noted.

The median [IQR] 5‐item compassion measure score was 16 (11–20). Five patients were missing a response to at least one 5‐item compassion measure item. The distribution of the 5‐item compassion measure scores is displayed in Figure S3. The 5‐item compassion measure ranged the full scale from 5 (lowest perceived compassion) to 20 (highest perceived compassion), and 27.0% (30/111) of respondents gave perfect scores (i.e. score of 20). The 5‐item compassion measure had excellent internal reliability among the included cohort (Cronbach's alpha = 0.92). We did not find patient or ED characteristics to be associated with patient experience of ED compassion (Table S3).

The median [IQR] fear of enacted stigma subscale score was 23 (16–31) and 97% (95% CI 91%–99%) reported some degree of stigma (i.e. score >10). Twelve patients were missing a response to at least one fear of enacted stigma subscale item. The distribution of the fear of enacted stigma subscale scores is displayed in Figure S4. The fear of enacted stigma subscale had good internal reliability among the included cohort (Cronbach's alpha = 0.86).

We found patient experience of ED compassion had a significant unadjusted negative association with patient fear of enacted stigma, β = −0.63 (95% CI −1.01 to −0.25). These results remained unchanged after adjusting for potential confounders (β = −0.66 [95% CI −1.03 to −0.29]; Table 3), suggesting that every 1‐point increase in the 5‐item compassion measure score is associated with a 0.66‐point decrease in the fear of enacted stigma score. Using structural equation modeling to allow patients with missing questionnaire data to be included we found similar results, β = −0.66 (95% CI −1.01 to −0.31; Table S4).

**TABLE 3:** Multivariable linear regression results.

Variables	β coefficients	95% CI
Patient experience of compassion^a^	−0.66	−1.03 to −0.29
Age (years)	0.03	−0.12 to 0.18
Polysubstance use	1.60	−3.02 to 6.23
IV opioid use	0.75	−2.70 to 4.20
Living in a shelter/on the street	−3.35	−7.10 to 0.41

*Note*: Fear of enacted stigma score as the dependent variable (pairwise analysis, *n* = 103).

^a^ Every 1‐point increase in the 5‐item compassion measure composite score.

**FIGURE 2:**
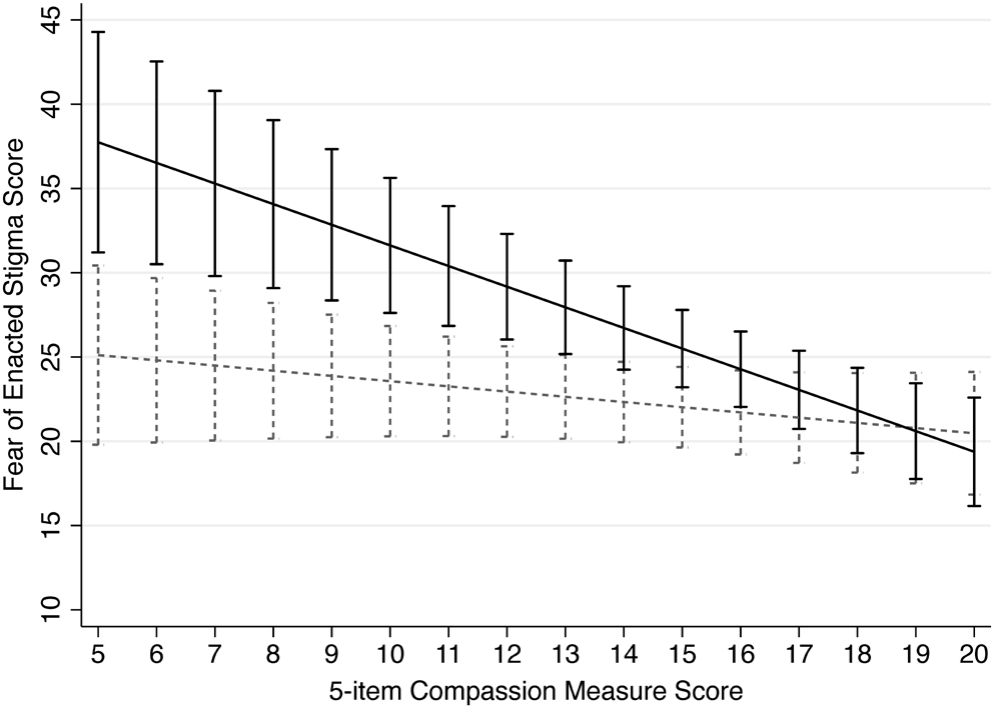
Linear prediction of patient experience of ED compassion (5‐item compassion measure) on fear of enacted stigma among patients presenting to the ED for an opioid‐specific problem (black, solid line) and patients with OUD presenting primarily for a non–opioid‐related problem (gray, dashed line). Adjusted for patient age (years), polysubstance use, IV opioid use, and living in a shelter/on the street. Vertical bars represent the 95% CIs. OUD, opioid use disorder.

On our sensitivity analysis we found patients who presented for an opioid‐specific problem had significantly higher fear of enacted stigma compared to patients with OUD presenting primarily for a non–opioid‐related problem, β = 18.02 (95% CI 5.66–30.37; Figure 2). We also found the interaction term between patient experience of compassion and presentation for an opioid‐specific problem to be statistically significant (Table 4). These results suggest the association between patient experience of ED compassion and fear of enacted stigma among patients presenting for an opioid‐specific problem (β = −1.22 [95% CI −1.79 to −0.66]) is stronger than among patients presenting primarily for a non–opioid‐related problem (β = −0.31 [95% CI −0.81 to 0.19]).

**TABLE 4:** Multivariable linear regression results.

Variables	β Coefficients	95% CI
Patient experience of compassion^a^	−0.31	−0.81 to 0.19
ED presentation for opioid‐specific problem	18.01	5.66 to 30.37
Interaction term	−0.92	−1.68 to −0.15
Age (years)	0.04	−0.11 to 0.20
Polysubstance use	1.56	−2.91 to 6.03
IV opioid use	−1.47	−5.25 to 2.31
Living in a shelter/on the street	−4.33	−7.65 to −1.02

*Note*: Fear of enacted stigma score as the dependent variable, entering an interaction term between patient experience of compassion and ED presentation for an opioid‐specific problem (pairwise analysis, *n* = 103).

^a^ Every 1‐point increase in the 5‐item compassion measure composite score.

## DISCUSSION

In this cross‐sectional study, we found that almost all patients reported experiencing fear of enacted stigma, and the minority of patients reported a perfect score on the 5‐item compassion measure. We found patient experience of greater compassion in the ED was an independent predictor of lower fear of enacted stigma. We also found that patients presenting for an opioid‐specific problem reported significantly greater fear of enacted stigma than patients presenting for a non–opioid‐related problem, and experience of greater compassion in the ED had a stronger association with lower fear of enacted stigma among patients presenting for an opioid‐specific problem.

Patients with OUD have reported degrading interactions with health care professionals, which negatively impact engagement in risk reducing behaviors.^32^ A study published in 2021 found that patients with OUD felt they were viewed as ‘morally culpable, intimidating, curious, untrustworthy and less valuable than other patients’ by health care workers.^12^ Negative attitudes towards this patient population likely results in these patients experiencing less compassion. The proportion of patients in this study that reported a perfect compassion score was approximately half that of prior studies using the 5‐item compassion measure to measure compassion in the same academic medical center ED (56% perfect score)^23^ and in the inpatient setting across 91 U.S. hospitals (54% perfect score)^21^ among cohorts that were not specific to patients with OUD. Our results did not find that wait time or being evaluated in a hallway versus private room were associated with patient experience of compassion, indicating that ED staff–patient interactions are more important drivers of patient experience of compassion than these external factors. This is consistent with prior research that has identified that simple interventions such as sitting (vs. standing) during the patient interview; eye contact; and verbal statements of acknowledgment, validation, and support may increase patient experience of compassion.^33^ However, it is unclear whether training physicians to routinely perform these actions can increase engagement in addiction treatment by reducing fear of enacted stigma, and further research is warranted.

Prior research has found racial disparities in OUD treatment.^34^ A recent qualitative study identified a lack of compassion and more experiences of stigma during medical care among Black patients decreased motivation to seek OUD treatment.^35^ While our study did not find a difference in compassion from ED staff between White and non‐White patients, our results do suggest that poor interactions during medical care are associated with fear of enacted stigma. Future research is warranted to examine the interaction of opioid‐related stigma and experiences of racial discrimination and its effect on engagement in addiction treatment.^36^

Prior studies of other patient populations have found greater compassion from health care providers is associated with better patient adherence to prescribed therapies, and conversely, a lack of clinician compassion is associated with nonadherence and more patients being lost to follow‐up.^37^, ^38^, ^39^ In this study we found that greater compassion from ED staff was associated with less fear of enacted stigma. Given that fear of enacted stigma in health care settings is associated with decreased health care–seeking behaviors among patients with OUD,^11^, ^12^ our results provide scientific rationale for future research to test if increasing compassion from ED staff decreases fear of enacted stigma and increases engagement in addiction treatment in this patient population.

Our observation that patients presenting for an opioid‐specific problem reported significantly greater fear of enacted stigma but had a stronger association between compassion from ED staff and lower fear of enacted stigma, suggests that patients presenting for an acute opioid complication may be in a highly vulnerable state and may be particularly sensitive to differences in compassion. Therefore, this specific population may be most receptive to interventions aimed at increasing patient experience of compassion.

## LIMITATIONS

We acknowledge that this study has important limitations to consider. First, this was an observational study and thus we can only report association rather than infer causation. While our study found an association between patient experience of compassion in the ED and fear of enacted stigma, future trials are needed to test whether interventions aimed at increasing compassion cause a decrease in stigma and increase engagement in addiction treatment. Second, assessment of patient experience of compassion may be subjective and influenced by factors external to the ED staff–patient interactions. However, our results did not find that sex, race, wait time, or being evaluated in a hallway were associated with patient experience of compassion. Also, to reduce the risk of recall bias, we prospectively measured patient experience of compassion at the end of patient care while patients were still in the ED. Third, we only enrolled English‐speaking subjects. Fourth, we performed a convenience sample when research associates were available to administer the research questionnaire in the ED. It is possible that patients presenting outside of the screening hours may experience compassion and/or stigma differently. Fifth, there is risk of nonresponse bias, with patients who declined to participate experiencing a different degree of compassion and/or fear of enacted stigma. It is reasonable to postulate that some patients declined to participate because they experienced lower compassion and higher fear of enacted stigma than those who participated, which may incite self‐protective hiding behavior; thus the degree of fear of enacted stigma in our cohort may be biased lower. Similarly, we only enrolled patients who presented to the ED for medical care. It is possible that patients who have greater fear of enacted stigma avoid presenting to the ED, resulting in this cohort underestimating fear of enacted stigma among patients with OUD. Further, given that we screened patients for a documented history of OUD or an opioid‐related complication, it is possible that patients with undiagnosed OUD were missed.

## CONCLUSIONS

In summary, this cross‐sectional study found that fear of enacted stigma is common among patients with opioid use disorder, and patient experience of greater compassion in the ED had a significant association with lower fear of enacted stigma. This association was stronger among patients presenting for an opioid‐specific problem. Future research is warranted to test whether interventions aimed at increasing compassion from ED staff reduce patient fear of enacted stigma and health care avoiding behaviors among patients with opioid use disorder.

## AUTHOR CONTRIBUTIONS

All authors have made substantial contributions to this paper: Brian W. Roberts supervised all aspects of the study and takes responsibility for the paper as a whole. Brian W. Roberts, Stephen Trzeciak, Brian M. Fuller, Michael B. Roberts, and Christopher W. Jones conceived this study. Rachel Haroz, Iris Jones, William Skelton, and Brian W. Roberts acquired the funding. Brian W. Roberts, Savannah Steinhauser, Christopher W. Jones, and William Skelton acquired the data. Brian W. Roberts, Christopher W. Jones, and Savannah Steinhauser managed the data. Brian W. Roberts, Savannah Steinhauser, and Michael B. Roberts analyzed the data and interpreted results. Brian W. Roberts and Savannah Steinhauser drafted the manuscript and all authors contributed substantially to its revision. All authors approved the final manuscript.

## FUNDING INFORMATION

Funded by the Camden Coalition, Pledge to Connect Initiative Grant.

## CONFLICT OF INTEREST STATEMENT

CWJ has no competing interests related to this work, though he has been an investigator on studies funded by AstraZeneca, Vapotherm, Abbott, and Ophirex. ST has co‐authored two books on compassion. He donates his book proceeds to the Cooper Foundation and has received payments for speaking engagements related to the books. The authors declare no conflicts of interest.

## Supporting information


Data S1:
